# Identifying barriers and policy priorities for rare disease research in underrepresented European countries

**DOI:** 10.1093/eurpub/ckag103

**Published:** 2026-06-13

**Authors:** Baiba Lace, Inna Inashkina, Dorica Dan, Ariane Weinman, Pauline Adam, Oleg Kvlividze, Birute Tumiene, Anabela Isidro, Carlos Almeida Pereira, Gonçalo Teixeira, Rachel K Crowley, ERDERA UC Stakeholder Panel, Dodo Agladze, Maya Atanasoska, Dimitrios Athanasiou, Aleksandra Augusciak-Duma, Olga Azevedo, Celia Azevedo Soares, Mehmet Cihan, Hana Boček, Lucimere Bohn, Hilmi Bolat, Joaquim Brites, Milos Brkusanin, Andra Ciuca, Senol Demir, Göksun Demirel, Murat Eyuboglu, Ozan Emre Eyupoglu, Naima Fdil, Laetitia Gaspar, Chahid Imane, Barbara Jenko Bizjan, Radka Kaneva, Peter Klivenyi, Piotr Kosla, Jernej Kovac, Gül Kozalak, Persefoni Kritikou, Gvantsa Kvantaliani, Sabine Laktina, Aleksandra Lesiak, João Lobo, Ausra Lukosiute-Urboniene, Milan Macek, Adriana Minovic, Rada Miskovic, Joanna Narbutt, Anabela Oliveira, Katrin Ounap, Sofia Ourani, Irem Ozgoren Kinli, Malgorzata Pawlowicz, Esra Serdaroglu, Sera Şimşek Derelioğlu, Karolina Śledzińska, Maja Stojiljkovic, Nino Nana Tatishvili, Tinatin Tkemaladze, Ivan Tourtourikov, Evrim Unsal, Athina Ververi

**Affiliations:** Department of Genetics, Riga East University hospital, University of Latvia, Riga, Latvia; Centre of Pathology, Riga East University hospital, Latvian Biomedical Research and Study Centre, Riga, Latvia; EURORDIS, Paris, France; EURORDIS, Paris, France; INSERM, Paris, France; Georgian Foundation for Genetic and Rare Diseases, Tbilisi, Georgia; Faculty of Medicine, Vilnius University, Vilnius, Lithuania; Agency for Clinical Research and Biomedical Innovation (AICIB), Porto, Portugal; Agency for Clinical Research and Biomedical Innovation (AICIB), Porto, Portugal; Agency for Clinical Research and Biomedical Innovation (AICIB), Porto, Portugal; Rare Disease Clinical Trial Network, University College Dublin (UCD), Dublin, Ireland

## Abstract

The European Joint Programme on Rare Diseases successfully advanced rare disease research and also revealed challenges for underrepresented countries, those less frequently holding or leading grants. This study aimed to survey Rare Disease researchers in these countries, identify barriers to participation in research, and propose solutions. A modified Delphi approach without formal consensus thresholds of 186 respondents highlighted fragmented or outdated policies and heterogeneous funding. Nearly all participants prioritized the need for EU-wide policies defining minimum quality standards for Rare Disease care. Key priorities include access to genetic testing and essential services to support uniform care and shared research capacity.

## Introduction

The formation of the European Union (EU) represents one of the most important economic and political achievements in modern Europe. Egalitarianism remains a moral cornerstone of democratic policy within the EU. However, certain disparities persist across Member States. One notable area of inequality concerns access to and success in EU research funding. Several countries rarely act as EU project leaders, and their overall success rates in securing EU grants are limited. Additionally, national research funding allocation within EU countries also differs substantially. As O. Petersen notes, the science budgets of Denmark, Finland, and Germany are up to ten times higher than those of certain Eastern European countries. Consequently, scientists operating within disproportionately smaller research budgets must compete in EU grant calls under structurally unequal conditions, often with limited prospects of success. This imbalance contributes to brain drain and/or to a gradual withdrawal of researchers from scientific activity in many countries, further deepening disparities [1].

This issue is strongly reflected in the field of Rare Diseases (RD). Resource-intensive RD care mirrors the same challenges seen in research access, and also affects patient care and treatment availability [2, 3]. In 2019, the European Joint Programme on Rare Diseases (EJPRD) was established with the purpose of improving the lives of RD patients through comprehensive research ecosystems. While the initiative has achieved important progress, it has also been overshadowed by the under-representation of several countries among EU grant recipients, often referred to as “underrepresented countries (UC)” [4], a definition applied to countries with limited visibility, participation, or influence in global systems. In this study, “underrepresented countries” refers to a programme-defined category used within ERDERA to describe countries with historically low participation and leadership rates in EU-funded rare disease research, particularly Joint Transnational Calls. This grouping is operational rather than socio-economic and includes both EU Member States and associated third countries participating in ERDERA activities. The present analysis treats this group as a shared policy-relevant context rather than a homogeneous analytical population. Among the UCs are Bulgaria, Cyprus, Czechia, Estonia, Greece, Georgia, Hungary, Ireland, Latvia, Lithuania, Morocco, Poland, Portugal, Romania, Serbia, Slovakia, Slovenia and Türkiye [5]. To seek solutions to this complex challenge, the Horizon Europe Initiative ERDERA—European Rare Diseases Research Alliance, launched in 2024, designed and is actively implementing a Work Package (WP 24 «Fostering engagement of underrepresented countries in ERDERA»), specifically aimed at supporting actions to improve UC participation in all ERDERA activities, including Joint Transnational Calls, Networking Support Scheme, and other specific initiatives.

The aim of this study was to conduct a survey of the RD community in UC (including medical specialists, researchers, and patients) to identify the major issues limiting successful participation in research projects, as well as to gather self-reported suggestions for potential solutions.

## Methods

Experts identified from UCs were interviewed, and the themes identified were reviewed within ERDERA WP24 to form a draft of the first survey. The first survey was disseminated through ERDERA emails and social media and also shared directly with a contact list of 650 rare disease experts from 18 UCs maintained by ERDERA WP24 (researchers, clinicians, policymakers, and patients).

The survey followed a modified Delphi-informed design. Participants who completed Phase I (186) were subsequently invited by email to participate in Phase II. Phase II was completed by 64 participants (34% of invitees).

Phase I was exploratory and aimed to elicit a broad range of barriers and priorities through open- and closed-ended questions. Responses were thematically grouped by the study team within ERDERA WP24 and consolidated into discrete problem and solution statements relevant to the RD community across the following themes: country profile, policy, collaboration, funding, technical assistance, and patient engagement. Phase II focused on priority ranking of these consolidated items (ranked items listed in Supplement 2). No formal consensus threshold was predefined; the objective was prioritization rather than consensus generation.

### Statistical analysis

Phase II results were analysed using the Borda count method. In addition to ranking the Phase I results, exploratory statistical analyses were performed using χ^2^ tests and Fisher’s exact test, with a significance threshold of *P* < .05 to identify statistically significant associations between the funding and service profiles of the UC as per participant responses and the grant participation reported. All country-level variables were self-reported by respondents and were not independently validated against external datasets.

## Results

In both survey phases all 18 UCs were represented. The proportion of responses in phase II was as follows: Turkey 13; Portugal 11; Poland 6; Serbia 6; Czech Republic 4; Georgia 4; Bulgaria 3; Greece 3; Slovenia 2; Morocco 2; Latvia 2; Lithuania 2; Ireland 1; Romania 1; Hungary 1; Estonia 1; Cyprus 1; Slovakia 1 [5]. These respondents ranked the most important problems and solutions proposed in phase I, framed by their own opinion of their own national environment and the international context. Phase II responses were unevenly distributed across countries, reflecting a purposive and convenience sample rather than proportional national representation.

Table 1 displays the five responses for the most identified/scores problems and five responses for the proposed solutions. The specific problem and solution statements ranked are shown in Supplement 2.

**Table 1.** Ranked problems and solutions obtained from Phase I survey, of 10 questions (ranked items listed in Supplement 2)

Problems	Borda score
Fragmented national strategies: Many countries lack a cohesive rare disease policy or have outdated plans	165
Smaller countries have only a limited number of rare disease specialists and/or centres. These specialists often cover multiple areas, leaving little time for focused research.	154
Heterogeneous healthcare funding: In some countries, advanced diagnostic technologies are either not reimbursed or results are delayed, limiting patient care and slowing research progress. This also prevents these countries from being leading partners in consortia.	147
Unequal access to EU programmes: Smaller institutions or patient groups often lack capacity to join large consortia, which may be reluctant to include small partners with limited funding.	139
Limited representation: Few countries have national representatives with rare disease expertise at the EU level, resulting in weak lobbying at higher institutions.	124
**Solutions**	
Develop a policy setting minimum quality requirements for rare disease patient care across Europe, including reimbursement for genetic testing and access to essential services. This would enable uniform care, guideline development, and a shared research platform.	220
Create dedicated programmes and grants for rare disease research at EU and national levels.	214
Provide training in project management, regulatory compliance (bioethical, data management), and novel methodologies (available research innovation techniques and facilities).	209
Establish patient advisory boards and include patient advocates as equal partners.	203
Create a uniform platform to identify all available patient registries and biobanks within the EU.	200

Of note, although 14 UCs have a Rare Disease national plan, 29% are not reported to be operational. Additionally, Phase I survey responses revealed that participants reporting the existence of a national rare disease plan with research components also tended to report the presence of registries and/or biobanks (χ^2^ tests, *P* < .001). Countries with a national RD plan that included research components were significantly more likely to report the existence of registries and/or biobanks and to report successful funding applications (χ^2^ tests, *P* < .01).

## Discussion

Respondents of UC ranked RD policy fragmentation as one of the most important problems. Mechanisms to ensure successful implementation of RD plans within the planned timeframe are often absent.

The impact of erratic provision and timing of research funding was noted by the ERDERA WP. The survey results raised concern that in the absence of operational RD plans and with irregular research funding, there is the potential for research funds to be redirected to patient care to address systemic healthcare limitations in introducing novel diagnostic technologies.

The lack of defined clinical and research budgets for rare disease generates secondary challenges, including the absence of functional national and cross-border networks.

Some UCs have small population sizes, which leads to a lack of critical mass of experts and results in a “multiple hats” effect, where a single person assumes multiple clinical, research and advocacy roles. This situation does not allow for effective or sustainable research capacity in narrow areas of RD research. Administrative insufficiencies that limit engagement in existing networks exacerbate the lack of local expertise to further reduce the capacity to submit high-quality, large-scale research applications within the RD ecosystem.

Support for patient organizations remains largely token rather than operational or impactful. Patients and their representatives are not compensated for participation in patient advisory boards, despite the demanding workload and the high level of expertise required. Patient advisory boards should be established and supported financially at all levels of decision-making.

In response to these challenges, respondents overwhelmingly prioritized the development of a policy that establishes minimum quality requirements for RD patient care across Europe, including reimbursement for genetic testing and guaranteed access to essential services. Such a framework would enable more uniform care, facilitate guideline development, and promote a shared research platform.

The survey highlights the challenges faced by RD medical communities in UC countries, which often struggle to exert sufficient influence on national regulatory bodies. Respondents suggested that European-level accreditation requirements could identify critical gaps and ensure the highest possible quality of care for patients.

Survey participants from UCs with a functional national RD plan—including research, registries, and training—reported better performance in European research funding competitions and clinical trial enrolment. Registries that were not accessed for trial enrolment or lacked interoperability with international partners were not reported to have value for patients or clinicians. National RD plans should therefore support research-oriented and interoperable registry and biobank infrastructures. These associations should be interpreted cautiously, as they reflect respondent perceptions of national infrastructure rather than independently verified system indicators. Given the heterogeneity of the UC group, findings should be interpreted as identifying common structural themes rather than country-specific system characteristics.

Survey respondents identified a need for research training in all aspects of rare disease research and research management, which should also be supported by national RD strategies.

As a limitation, while all UC countries were represented, the findings primarily reflect the perspectives of a purposively sampled and unevenly distributed respondent group and cannot be considered representative of all rare disease stakeholders in each country. Although some heterogeneity was expected, respondents demonstrated a remarkably consistent identification of key problems and potential solutions.

## Supplementary Material

ckag103_Supplementary_Data

## Data Availability

The data underlying this article are available in the article and in its online supplementary material.

## References

[1] Petersen OH. Inequality of research funding between different countries and regions is a serious problem for global science. Function (Oxf) 2021;2:zqab060. 10.1093/function/zqab060 35330794 PMC8788724

[2] Kluszczynski T , Nemeth B, Władysiuk M et al Optimizing patient access to orphan medicinal products: lessons from Central and Eastern Europe. J Mark Access Health Policy 2025;13:24. 10.3390/jmahp13020024 40568380 PMC12194612

[3] Kostadinov K , Iskrov G, Musurlieva N et al ‘It felt like finding hope only to lose it again’: a grounded theory study of rare cancer policies in Bulgaria. J Cancer Policy 2025;44:100570. 10.1016/j.jcpo.2025.100570 40081491

[4] Mimouni Y , Halftermeyer J, Petton Y et al The European joint programme on rare diseases: building the rare diseases research ecosystem. Rare Dis Orphan Drugs J 2024;3:2–10. 10.20517/rdodj.2024.06.

[5] Although Morocco and Georgia are not EU Member States, they participate in ERDERA as partner countries. Their involvement reflects the EU’s approach of promoting regional cooperation and knowledge exchange with associated third countries.

