# Supplementary material for: Identifying barriers and policy priorities for rare disease research in underrepresented European countries
Source: Eur J Public Health. 2026 Jun 13;36(4):ckag103. doi: 10.1093/eurpub/ckag103 (PMC13265805; doi:10.1093/eurpub/ckag103)
Supplement: ckag103_Supplementary_Data [file ckag103_supplementary_data.zip › ejph-2026-04-sr-0372-File003.docx]

IDENTIFYING BARRIERS AND POLICY PRIORITIES FOR RARE DISEASE RESEARCH IN UNDERREPRESENTED EUROPEAN COUNTRIES

Supplement 1.

**ERDERA UC Stakeholder Panel**

**Complete list of authors (same credit as main list)**

1. Dodo Agladze, Georgian foundation for genetic and rare diseases (GERAD), Georgia
2. Maya Atanasoska, GMDL Cell genetics, Bulgaria
3. Dimitrios Athanasiou, Enosi spanion asthenon ellados (RDG), Greece
4. Aleksandra Augusciak-Duma, Medical University of Silesia (SUM), Poland
5. Olga Azevedo, Hospital Senhora da Oliveira – Guimarães, Portugal
6. Celia Azevedo Soares, Universidade de Aveiro, Aveiro, Portugal
7. Mehmet Cihan, Balcı Istanbul University, Turkey
8. Hana Boček, Fakultni Nemocnice v Motole (MUH), Czech Republic
9. Lucimere Bohn, University of Porto, Portugal
10. Hilmi Bolat, Balıkesir University, Turkey
11. Joaquim Brites, APN - Associação Portuguesa de Neuromusculares, Portugal
12. Milos Brkusanin, University of Belgrade-Faculty of Biology, Serbia
13. Andra Ciuca, Babes-Bolyai University, Romania
14. Senol Demir, Van Research and Training hospital, Turkey
15. Göksun DEMİREL, Turkiye Bilimsel Ve Teknolojik Arastirma Kurumu (TUBITAK), Turkey
16. Murat Eyuboglu, Eskisehir Osmangazi University Medical School, Turkey
17. Ozan Emre Eyupoglu, Istanbul Medipol University, Turkey
18. Naima Fdil, Universite Cadi Ayyad (CAU), Morocco
19. Laetitia Gaspar, Universidade De Coimbra (UC), Portugal
20. Chahid Imane, Université Hassan II De Casablanca (UNIVH2C), Morocco
21. Barbara Jenko Bizjan, Univerzitetni Klinicni Center Ljubljana (UKCL), Slovenia
22. Radka Kaneva, Medical University Sofia (MUS), Bulgaria
23. Peter Klivenyi, University of Szeged, Hungary
24. Piotr Kosla, PACS2 Research Foundation, Poland
25. Jernej Kovac, Univerzitetni Klinicni Center Ljubljana (UKCL), Slovenia
26. Gül Kozalak, Sabanci University, Turkey
27. Persefoni Kritikou, Rare Disease Consulting, Greece
28. Gvantsa Kvantaliani, Georgian Foundation For Genetic And Rare Diseases (GERAD), Georgia
29. Sabine Laktina, Bernu Kliniska Universitates Slimnica Valsts Sia (CCUH), Latvia
30. Aleksandra Lesiak, Medical University of Lodz, Department of Dermatology Pediatric Dermatology and Oncology MUL, Poland
31. João Lobo, Instituto Português de Oncologia do Porto (IPO PORTO), Portugal
32. Ausra Lukosiute-Urboniene, Lithuanian University of Health Sciences, Lithuania
33. Milan Macek, Fakultni Nemocnice V Motole (MUH), Czech Republic
34. Adriana Minovic, CRO, Serbia
35. Rada Miskovic, University Clinical Center of Serbia, Serbia
36. Joanna Narbutt, Medical University of Lodz, Departament of Dermatology, Pediatric Dermatology and Oncology, Poland
37. Anabela Oliveira, Adult Inherited Metabolic Diseases Center Santa Maria Hopital, Portugal
38. Katrin Ounap, Tartu Ulikool (UTARTU), Estonia
39. Sofia Ourani, Hospital Archbishop Makarios III, Cyprus
40. Irem Ozgoren Kinli, Izmir Katip Celebi University, Turkey
41. Malgorzata Pawlowicz, Regional Specialized Children's Hospital in Olsztyn, Pola
42. Esra Serdaroglu, Gazi University, Turkey
43. Sera Şimşek Derelioğlu, Atatürk University, Turkey
44. Karolina Śledzińska, Gdański Uniwersytet Medyczny (gumed*), Poland
45. Maja Stojiljkovic, Institut Za Molekularnu Genetiku I Geneticko Inzenjerstvo (IMGGE), Serbia
46. Nino Nana Tatishvili, Georgian Foundation For Genetic And Rare Diseases (GERAD), Georgia
47. Tinatin Tkemaladze, Department of Molecular and Medical Genetics, Tbilisi State Medical University, Georgia
48. Ivan Tourtourikov, Medical University Sofia (MUS), Bulgaria
49. Evrim Unsal, Mikrogen Genetic Diagnosis Laboratory and Yüksek İhtisas University Faculty of Medicine Clinical Genetics Department, Turkey
50. Athina Ververi, Dept of Genetics for Rare Diseases, Papageorgiou NHS Hospital, Thessaloniki, Greece
