# Supplementary material for: Identifying barriers and policy priorities for rare disease research in underrepresented European countries
Source: Eur J Public Health. 2026 Jun 13;36(4):ckag103. doi: 10.1093/eurpub/ckag103 (PMC13265805; doi:10.1093/eurpub/ckag103)
Supplement: ckag103_Supplementary_Data [file ckag103_supplementary_data.zip › ejph-2026-04-sr-0372-File004.docx]

IDENTIFYING BARRIERS AND POLICY PRIORITIES FOR RARE DISEASE RESEARCH IN UNDERREPRESENTED EUROPEAN COUNTRIES

**Supplement 2**

**Survey questions of phase II**

Policy Priorities

Problems – Rank from 1 to 4 according to your priorities:

1. Fragmented national strategies: Many countries lack a cohesive rare disease policy or have outdated plans.
2. Underrepresented countries: Smaller countries have only a limited number of rare disease specialists and/or centres. These specialists often cover multiple areas, leaving little time for focused research.
3. Limited representation: Few countries have national representatives with rare disease expertise at the EU level, resulting in weak lobbying at higher institutions.
4. Heterogeneous healthcare funding: In some countries, advanced diagnostic technologies are either not reimbursed or results are delayed, limiting patient care and slowing research progress. This also prevents these countries from being leading partners in consortia.

Solutions – Rank from 1 to 5 according to your priorities:

1. Increase the number of representatives from underrepresented countries at the EU expert level. Ensure national representatives have rare disease research expertise.
2. Develop a policy setting minimum quality requirements for rare disease patient care across Europe, including reimbursement for genetic testing and access to essential services. This would enable uniform care, guideline development, and a shared research platform.
3. Establish institutional or national support systems for EU grant proposal preparation.
4. Create dedicated programmes and grants for rare disease research at EU and national levels.
5. Launch ERDERA programmes for education and training in rare disease research.

Collaboration

Problems – Rank from 1 to 3 according to your priorities:

1. Lack of established networks: Researchers in underrepresented countries struggle to find partners for high-quality proposal preparation.
2. Unequal access to EU programmes: Smaller institutions or patient groups often lack capacity to join large consortia, which may be reluctant to include small partners with limited funding.
3. Language and administrative barriers.

Solutions – Rank from 1 to 3 according to your priorities:

1. Ensure grants for cross-border mentorship and knowledge exchange.
2. Set recruitment targets for diverse populations (ethnic, geographic, socioeconomic). Avoid exclusion criteria that disproportionately affect minority groups.
3. Develop international research infrastructures such as registries and biobanks that are easily accessible to all researchers and centralise information about them.

Funding

Problems – Rank from 1 to 3 according to your priorities:

1. Insufficient national funding: Many countries do not allocate specific budgets for rare disease research.
2. Bias in EU funding: Perceived favouritism towards applicants from certain countries or institutions.
3. Complex application processes: Time-consuming and resource-intensive, especially for smaller teams without Institutional support.

Solutions – Rank from 1 to 3 according to your priorities:

1. Increase number of grants for rare disease research and inclusive initiatives.
2. Provide grants for early-career researchers and underrepresented groups. Include inclusivity criteria in project evaluations and funding calls.
3. Allocate budgets for patient involvement and multilingual engagement.

Technical Assistance

Problems – Rank from 1 to 3 according to your priorities:

1. Lack of institutional support: Many researchers report no functional support system for proposal preparation.
2. Uneven distribution of expertise: Technical knowledge is often concentrated in a few centres.
3. Limited training opportunities: Especially in project management, regulatory compliance, and novel methodologies.

Solutions – Rank from 1 to 5 according to your priorities:

1. Provide training in project management, regulatory compliance (bioethical, data management), and novel methodologies (available research innovation techniques and facilities).
2. Develop e-consent platforms and telehealth recruitment tools. Ensure interoperable databases for secure data sharing.
3. Create a uniform platform to identify all available patient registries and biobanks within the EU.
4. Offer technical assistance for proposal preparation.
5. Establish matching mechanisms for complementary research groups.

Patient Engagement

Problems – Rank from 1 to 3 according to your priorities:

1. Patients are sometimes included symbolically rather than as true partners.
2. Lack of funding for patient organisations: Limits their ability to participate meaningfully.
3. Barriers to participation: Travel costs, digital access, and accessibility issues.

Solutions – Rank from 1 to 4 according to your priorities:

1. Establish patient advisory boards and include patient advocates as equal partners.
2. Define required tasks for patient organisations in research applications, such as ethical frameworks and consent processes, and in providing guidance to drive research towards unmet medical needs.
3. Provide trainings for patients, caregivers, patient organisations to participate in research projects.
4. Ensure to financially compensate the time of patient representatives as equal partners in projects. Provide travel grants dedicated to patient organisations to participate in relevant meetings.
